# Supplementary material for: The interactome of intact mitochondria by cross-linking mass spectrometry provides evidence for coexisting respiratory supercomplexes
Source: Mol Cell Proteomics. 2017 Dec 8;17(2):216–32. doi: 10.1074/mcp.RA117.000470 (PMC5795388; doi:10.1074/mcp.RA117.000470)
Supplement: Supplemental Data [file supp_RA117.000470_133922_0_supp_23978_fzffwf.zip › spectra_annotation/mito_DR_spectra_annotation/28-1-4-1-5-1.pdf]

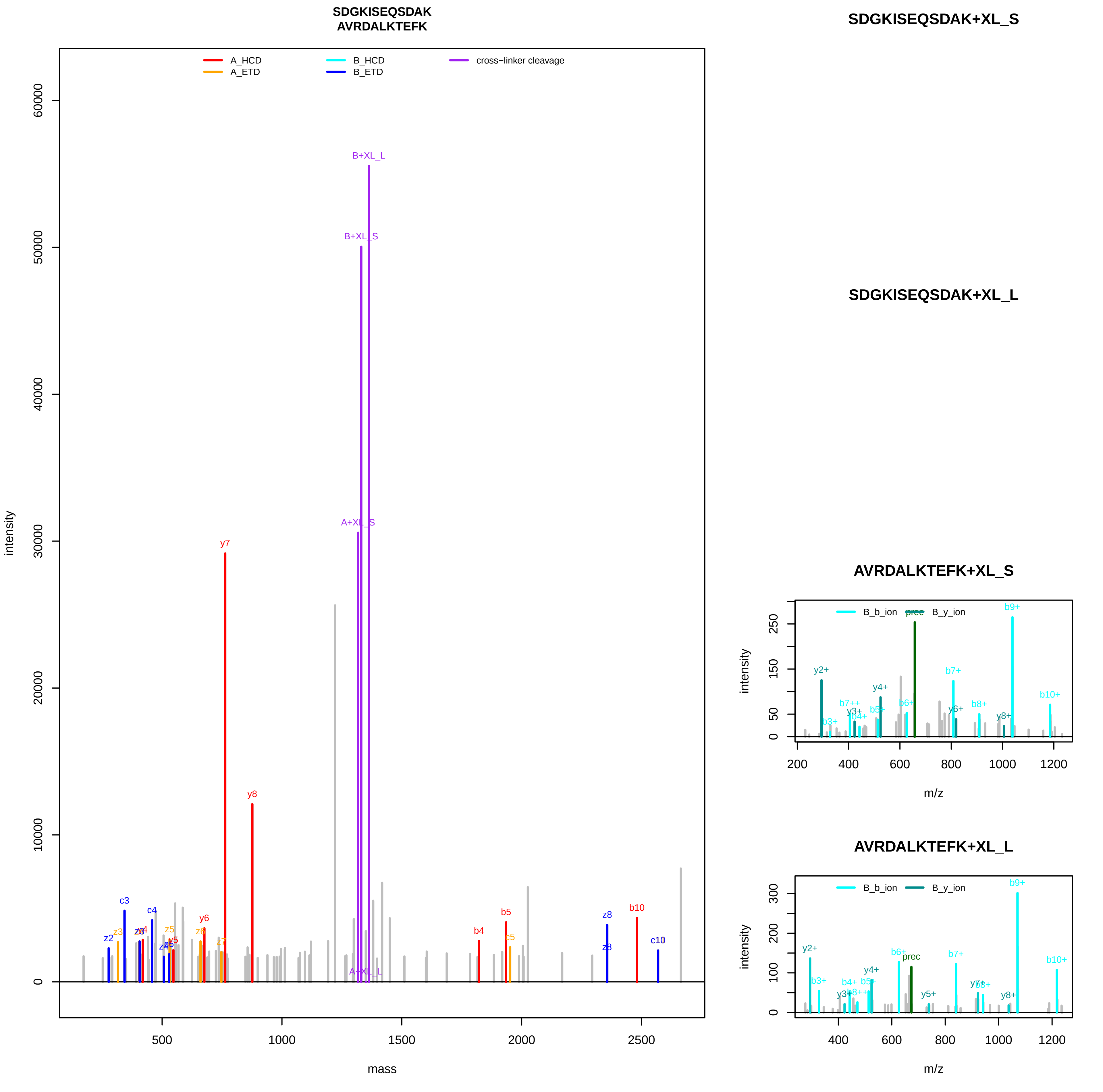

SDGKISEQSDAK
AVRDALKTEFK
A_HCD
A_ETD
B_HCD
B_ETD
cross−linker cleavage
B+XL_L
B+XL_S
A+XL_S
A+XL_L
y7
y8
y6
y5
y4
y3
z2
z3
c3
c4
z4
c5
z5
z6
z7
b4
b5
c5
z8
b10
c10
intensity
mass
SDGKISEQSDAK+XL_S
SDGKISEQSDAK+XL_L
AVRDALKTEFK+XL_S
B_b_ion
B_y_ion
prec
y2+
b3+
y3+
b7++
b4+
b5+
y4+
b6+
b7+
y6+
b8+
y8+
b9+
b10+
intensity
m/z
AVRDALKTEFK+XL_L
B_b_ion
B_y_ion
y2+
b3+
y3+
b4+
b8++
b5+
y4+
b6+
prec
y5+
b7+
y7+
b8+
y8+
b9+
b10+
intensity
m/z
